# The emerging oral microbe-host axis in endometriosis: a systematic review of *Fusobacterium nucleatum*

**DOI:** 10.3389/fcimb.2026.1994934

**Published:** 2026-10-16

**Authors:** Maria E. Ramos-Nino, Aniruddha Doshi, Prakash V. A. K. Ramdass

**Affiliations:** 1 Department of Microbiology, Immunology, and Pharmacology, School of Medicine, St. George’s University, St. George, Grenada; 2 Department of Public Health and Preventive Medicine, School of Medicine, St. George’s University, St. George, Grenada

**Keywords:** endometriosis, *Fusobacterium nucleatum*, *Fusobacterium*, oral microbiome, host–microbe interactions, fibrosis, systematic review

## Abstract

**Background:**

*Fusobacterium nucleatum*, an oral-associated bacterium implicated in several extraoral diseases, has recently been proposed as a potential contributor to endometriosis through microbial translocation, inflammation, and fibrosis. However, the consistency and clinical significance of this association remain uncertain. This systematic review evaluated the human and experimental evidence linking *F. nucleatum* with endometriosis and assessed the plausibility of a proposed oral microbe-host axis.

**Methods:**

PubMed/MEDLINE, Embase, Web of Science, and Google Scholar were searched from inception through August 31, 2026, following PRISMA 2020 guidelines. Human and animal studies reporting *F. nucleatum* or historically related *F. nucleatum*-complex taxa in relation to endometriosis were included. Risk of bias was assessed using a prespecified adapted ROBINS-I framework for human observational studies and SYRCLE for animal studies. Owing to substantial heterogeneity in anatomical compartments, detection methods, and outcomes, meta-analysis was not appropriate and findings were synthesized qualitatively.

**Results:**

Four human studies encompassing 384 participants at the study level were included, although analysis-specific denominators varied. Direct tissue evidence came from two studies and was conflicting: Muraoka et al. reported *Fusobacterium* positivity in 64.3% of endometrial samples from women with endometriosis versus 7.1% of controls in a 42-versus-42 tissue subset, whereas Graciano-España et al. found no significant case-control difference in *F. nucleatum* abundance among 55 cases and 37 controls. Indirect microbiome studies reported oral *Fusobacterium* enrichment in moderate/severe endometriosis and vaginal *F. animalis* depletion in chronic pelvic pain with endometriosis. All human studies were judged at serious overall risk of bias, and certainty of evidence was very low. Experimental evidence suggesting TGF-β-mediated lesion growth and fibrosis derived principally from one translational study and lacked independent replication.

**Conclusions:**

Current evidence supports biological plausibility but not a consistent or causal association between *F. nucleatum* and endometriosis. Direct tissue evidence is limited to two conflicting studies, while non-tissue microbiome findings are indirect. Standardized contamination-controlled sampling, paired anatomical-site sampling, strain-level analyses, and longitudinal studies are required before microbial translocation, temporality, causality, or antimicrobial treatment can be established.

**Systematic review registration:**

https://www.crd.york.ac.uk/PROSPERO/view/CRD420261337885.

## 1 Introduction

Endometriosis is a chronic gynecologic disorder characterized by endometrial-like tissue outside the uterus and affects approximately 190 million women worldwide (Zondervan et al., 2018). Common manifestations include chronic pelvic pain, dysmenorrhea, dyspareunia, and infertility, with substantial effects on quality of life and daily functioning (Becker et al., 2022). Despite extensive investigation, its pathogenesis remains incompletely understood and is likely multifactorial (Treloar et al., 2010; Peterson et al., 2013; Farland et al., 2017; Harris et al., 2018). Proposed mechanisms include retrograde menstruation, hematogenous or lymphatic dissemination, altered immune responses, and hormonal, genetic, and environmental factors (Sampson, 1927). Increasing attention has also focused on the potential contribution of microorganisms to endometriosis. The emerging concept of an “endobiota” challenges the traditional view of the upper reproductive tract as sterile and raises the possibility that microbial communities or specific pathobionts may influence local inflammation, immune dysregulation, and tissue remodeling (Ata et al., 2019). However, investigation of the reproductive tract microbiome remains challenging because endometrial and endometriotic tissues are low-biomass specimens highly susceptible to contamination during sampling and molecular analysis (Kaluanga Bwanga et al., 2023).

Among the microorganisms attracting particular interest is *F. nucleatum*, a Gram-negative anaerobe predominantly associated with the oral cavity and periodontal disease (Yang et al., 2025). Beyond its established oral niche, *F. nucleatum* has been implicated in extraoral diseases, particularly colorectal cancer, and emerging evidence suggests a potential link between oral microbiota and female reproductive health (Kostic et al., 2013; Rubinstein et al., 2013; Marcickiewicz et al., 2025). Its capacity for dissemination beyond the oral cavity is supported by its detection at distant anatomical sites, including placental tissues in adverse pregnancy outcomes (Han, 2015). These observations have raised the possibility of an oral microbe-host axis through which oral-associated *Fusobacterium* may disseminate to or influence distant tissues (Li et al., 2025). Whether such an axis contributes to endometriosis remains uncertain, however, and detection of *Fusobacterium* outside the oral cavity does not establish oral origin, persistent colonization, or causality (Wu et al., 2026).

Interest in this hypothesis increased following the translational study by Muraoka et al. in 2023, which reported increased *Fusobacterium* detection in endometrial and endometriotic tissues from Japanese women and provided experimental evidence of a potential host response (Muraoka et al., 2023). In a mouse model*, F. nucleatum* exposure promoted endometriotic lesion growth and TGF-β-associated fibrosis, while antibiotic treatment attenuated these effects (Muraoka et al., 2023). Subsequent human studies, however, have produced inconsistent findings. Graciano-España et al. found no significant difference in *Fusobacterium* spp. or *F. nucleatum* abundance in eutopic endometrium from women with and without endometriosis (Graciano-España et al., 2025). Evidence from other gynecologic conditions nevertheless supports the ability of *F. nucleatum* to occur within the female reproductive tract, with Hakimjavadi et al. reporting significantly greater vaginal abundance of *F. nucleatum* in women with high-grade endometrial cancer than in those with benign gynecologic disease (Hakimjavadi et al., 2022).

Collectively, these findings suggest a biologically plausible but unresolved relationship between oral-associated *Fusobacterium*, host responses, and endometriosis. Major uncertainties remain regarding the consistency and anatomical specificity of bacterial detection, microbial origin and routes of dissemination, associated inflammatory and fibrotic responses, and whether *Fusobacterium* represents a causal contributor, a consequence of the disease microenvironment, or an incidental microbial signal. This systematic review therefore aimed to critically synthesize the available human and experimental evidence linking *F. nucleatum* to endometriosis, evaluate the methodological quality and certainty of the evidence, and assess the biological plausibility and clinical implications of a proposed oral microbe-host axis.

## 2 Materials and methods

### 2.1 Protocol registration and reporting guidelines

This review was conducted in accordance with the PRISMA 2020 guidelines (Page et al., 2021). Eligibility criteria, risk-of-bias assessment, and synthesis methods were defined *a priori*. No methodological changes were made during the review, except for extending the GRADE framework to animal and mechanistic outcomes to allow appraisal across different evidence types (Guyatt et al., 2008). This review was prospectively registered in PROSPERO (CRD420261337885).

### 2.2 Search strategy

A comprehensive literature search was conducted in PubMed/MEDLINE, Embase, Web of Science Core Collection, and Google Scholar from database inception to August 31, 2026. Search strategies combined controlled vocabulary (MeSH in PubMed and Emtree in Embase) with free-text terms, including “*Fusobacterium nucleatum*” and the genus-level term “*Fusobacterium*,” combined with “endometriosis” and “endometrioma,” using Boolean operators. Database-specific syntax was applied as appropriate, and Google Scholar was searched using *Fusobacterium nucleatum* and endometriosis, with “all the words in the title.” Because the genus-level term Fusobacterium was included, records indexed under *F. animalis* remained retrievable; however, “*Fusobacterium animalis*” was not specified as a separate free-text term in the original strategy, which is acknowledged as a limitation.

### 2.3 Eligibility criteria and study selection

Eligible studies were peer-reviewed primary research articles published in English from database inception through August 31, 2026, that reported *F. nucleatum* in patients with endometriosis or relevant animal models. For the purposes of this review, *Fusobacterium animalis* was considered within the historically related *F. nucleatum* species complex because it was previously classified as *F. nucleatum subsp. animalis*. Although genome-based analyses proposed its reclassification as a distinct species in 2017, the species-level designation *F. animalis* was formally validated in 2022 (Munson et al., 2023). Accordingly, studies reporting *F. nucleatum subsp. animalis* or *F. animalis* were considered relevant to account for changes in bacterial nomenclature over time.

Human studies were required to provide quantitative or statistical comparisons between endometriosis cases and controls. Reviews, commentaries, conference abstracts without full-text publication, and studies reporting only broad microbiome findings without *Fusobacterium-*specific data were excluded. Eligible detection methods included fluorescence *in situ* hybridization, quantitative PCR, 16S rRNA sequencing, or other validated approaches. The presence of contamination controls was considered during risk-of-bias assessment but was not required for inclusion; studies without explicit contamination controls were retained when otherwise eligible and downgraded where appropriate. Two reviewers (M.N. and A.D.) independently screened titles and abstracts and subsequently assessed potentially eligible full-text articles using the same predefined criteria. Any disagreements were resolved by a third reviewer (P.R.).

### 2.4 Data extraction

Data were independently extracted by two reviewers (M.N. and A.D.) using standardized forms. Information collected included study design, population characteristics, sample type and collection methods, *Fusobacterium* detection methods, quantitative findings, contamination controls, clinical associations, and mechanistic outcomes. Extracted data were cross-checked to ensure accuracy and consistency with the predefined protocol. The primary outcome was the detection or abundance of *F. nucleatum* in endometrial or endometriotic tissue. Secondary outcomes included detection or enrichment of *Fusobacterium* taxa in oral and other biological samples, measures of abundance or association, and experimental outcomes from animal models. Mechanistic findings related to lesion development, fibrosis, and inflammatory signaling were also extracted when available.

### 2.5 Risk of bias assessment

Risk of bias in human observational studies was assessed using a prespecified adapted ROBINS-I framework (Sterne et al., 2016). The framework was used to structure judgments concerning confounding, participant selection, classification of endometriosis status, missing data, outcome measurement, and selective reporting; deviations from intended interventions/exposures were considered not applicable to purely observational studies. ROBINS-E was considered during revision because it was developed specifically for causal exposure-effect estimates; however, the current full ROBINS-E tool is designed for non-randomized follow-up studies of exposure effects (Higgins et al., 2024), whereas the included studies comprised heterogeneous cross-sectional, case-control, and cohort association designs and did not uniformly estimate a longitudinal causal exposure effect. We therefore retained the prespecified framework rather than switching tools *post hoc*. This methodological choice is acknowledged as a limitation, and future etiologic reviews based on longitudinal exposure-effect studies should preferentially consider ROBINS-E.

Animal studies were assessed using the SYRCLE Risk of Bias tool (Hooijmans et al., 2014), which evaluates potential bias related to sequence generation, baseline characteristics, allocation concealment, random housing, blinding, random outcome assessment, incomplete outcome data, and selective reporting.

### 2.6 Certainty assessment

The GRADE framework (Guyatt et al., 2008) was adapted to assess certainty of evidence across human, animal, and mechanistic outcomes. Certainty was evaluated according to risk of bias, inconsistency, indirectness, imprecision, and publication bias, with ratings ranging from high to very low.

### 2.7 Data synthesis

Meta-analysis was not performed because the included studies differed substantially in biological compartment, study design, microbial target, detection method, and reported effect measure. A qualitative synthesis was therefore conducted and organized by evidentiary proximity: direct tissue-based evidence from endometrial/endometriotic specimens was considered separately from indirect non-tissue microbiome evidence from oral, vaginal, rectal, and stool samples. Experimental animal findings were synthesized as mechanistic evidence rather than pooled with human observational findings.

### 2.8 Declaration of generative artificial intelligence use

During the preparation of this manuscript, the authors used generative artificial intelligence (ChatGPT-5.5, 2026; OpenAI), FigureLabs AI (August 2025), and Perplexity AI (free version) to assist with formatting, English language editing, clarification of text, and the creation and enhancement of graphical elements, including figures and illustrations, to improve their visual clarity and presentation. All scientific content, study design, data collection, analyses, interpretation of results, and manuscript conclusions were developed, verified, and approved by the authors. The authors take full responsibility for the accuracy and integrity of the work. All AI-assisted outputs were carefully reviewed, edited, and validated by the authors, who assume full responsibility for the accuracy, integrity, and content of this publication.

## 3 Results

### 3.1 Study selection

The database searches identified 35 records: 13 from PubMed, 6 from Web of Science, 13 from Embase, and 3 from Google Scholar. After removing 13 duplicates, 22 unique records underwent title and abstract screening, of which 10 were excluded. The remaining 12 articles were assessed in full text. Eight were excluded: 2 did not report *Fusobacterium* at the genus or species level, 2 were editorial letters, and 4 were review articles. Ultimately, 4 studies (Muraoka et al., 2023; Jimenez et al., 2024; Graciano-España et al., 2025; Hicks et al., 2025) met the eligibility criteria and were included in the systematic review. The PRISMA flow diagram of the study selection process is shown in **Figure 1**.

**Figure 1:**
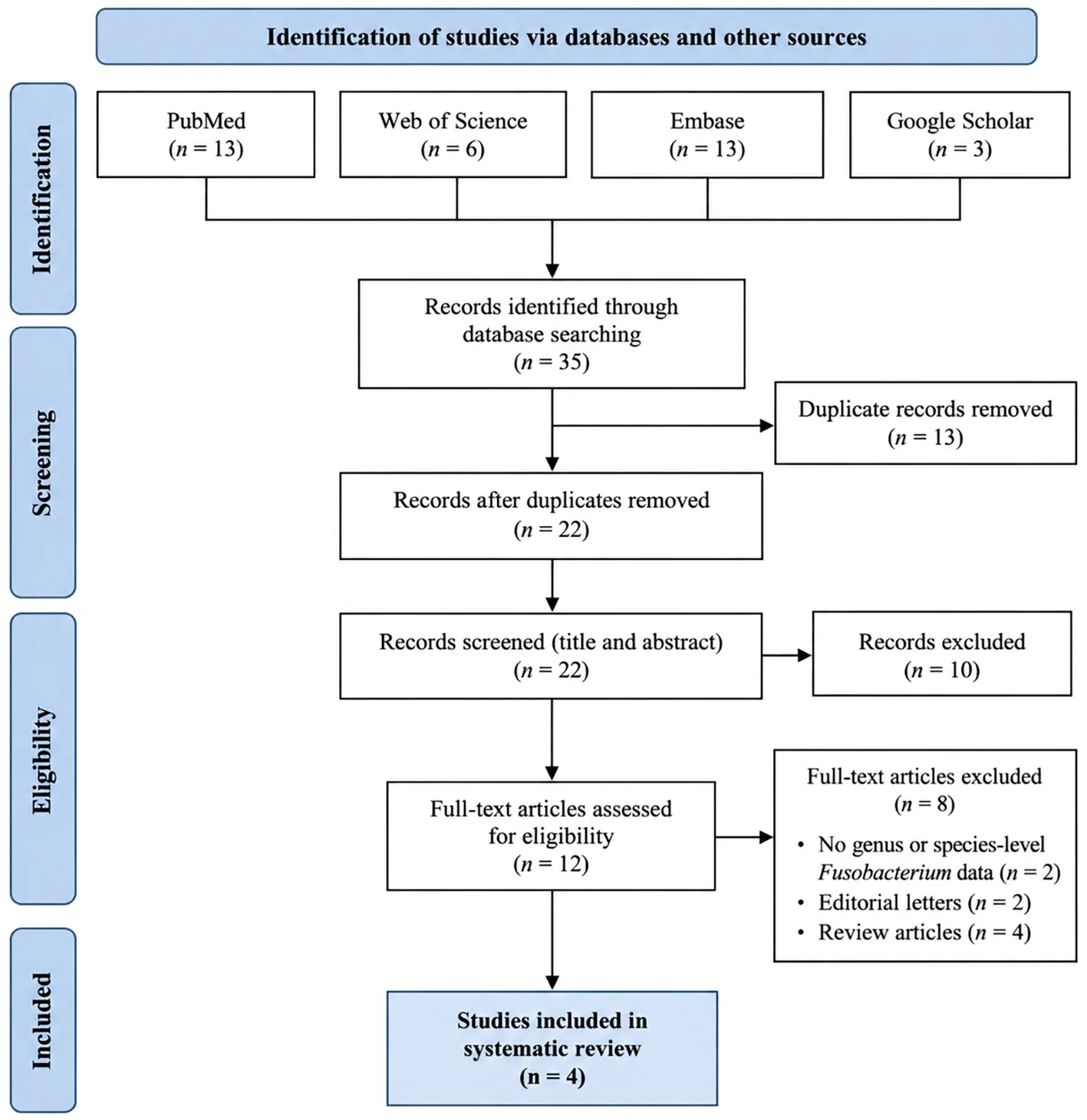
PRISMA flow diagram of the study selection process.

### 3.2 Study characteristics

Four studies published between 2023 and 2025 were included. The study populations were drawn from Japan, the United States (two studies), and Australia. At the study level, the four reports encompassed 384 participants: Muraoka et al. enrolled 79 women with endometriosis and 76 controls (n=155), although the principal tissue FISH comparison used a 42-versus-42 subset; Jimenez et al. reported a 73-participant three-group comparison; Graciano-España et al. included 55 endometriosis cases and 37 controls (n=92); and Hicks et al. included 21 confirmed endometriosis participants, 24 confirmed non-endometriosis participants, and 19 healthy controls (n=64). Because several analyses used subsets of the full study populations, outcome-specific denominators are reported separately in **Table 1**. Study designs included translational cross-sectional, retrospective case-control, prospective cohort, and pilot observational approaches, with one study also incorporating an experimental mouse model. Biological samples included endometrial tissue, endometriotic lesions, oral samples, vaginal swabs, rectal swabs, and stool samples. *Fusobacterium* was assessed using fluorescence *in situ* hybridization (FISH), quantitative PCR (qPCR), or 16S rRNA sequencing. This variation highlights the substantial methodological heterogeneity of the available evidence.

**Table 1:** Characteristics of included studies.

Study	Study setting	Design	Study groups/n	Sample(s)	Method	Key findings
Muraoka 2023 (Muraoka et al., 2023)	Japan	Translational cross-sectional + mouse model	Endometriosis n=79; controls n=76 (total n=155). Principal tissue FISH comparison: 42 vs 42.	Eutopic endometrium, endometriotic lesions; vaginal swabs	FISH; qPCR	*Fusobacterium* detected in 64.3% of endometrial samples from the 42-case subset vs 7.1% of 42 controls; 52.4% of endometriotic lesions positive. Mouse experiments: increased lesion growth/fibrosis; antibiotic attenuation.
Jimenez 2024 (Jimenez et al., 2024)	USA	Pilot observational	CPP-Endo n=35; CPP without endometriosis n=23; surgical controls n=15 (n=73 in final comparison)	Vaginal and rectal	16S rRNA (V4)	*F. animalis* depleted in CPP-Endo vs CPP without endometriosis (LFC = -0.63). No endometrial or endometriotic tissue assessed.
Graciano-España 2025 (Graciano-España et al., 2025)	USA (University of Pennsylvania Endomarker cohort)	Retrospective case-control	Endometriosis n=55 (42 minimal/mild; 13 moderate/severe); controls n=37 (n=92)	Eutopic endometrium	qPCR; genus- and species-specific primers	No significant difference in *Fusobacterium* spp. (P = .258) or *F. nucleatum* (P = .738) abundance between cases and controls.
Hicks 2025 (Hicks et al., 2025)	Australia	Prospective cohort pilot	Endometriosis n=21; confirmed no endometriosis n=24; healthy controls n=19 (n=64)	Oral, vaginal, stool	16S rRNA sequencing	Oral *Fusobacterium* enriched in moderate/severe vs minimal/mild endometriosis; no evidence of reproductive-tissue colonization or oral-to-tissue translocation.

FISH, fluorescence *in situ* hybridization; CPP, chronic pelvic pain; LFC, log fold change; qPCR, quantitative polymerase chain reaction.

### 3.3 Qualitative synthesis of evidence

#### 3.3.1 Direct tissue-based evidence

The two studies providing direct human evidence from endometrial or endometriotic tissue reported conflicting findings. Muraoka et al. (2023) reported the most pronounced association. In the principal tissue comparison*, Fusobacterium* was detected in 27 of 42 endometrial samples from women with endometriosis (64.3%) compared with 3 of 42 controls (7.1%) and in 22 of 42 endometriotic lesions (52.4%) (Muraoka et al., 2023). Complementary mouse experiments showed that *F. nucleatum* exposure increased lesion weight and promoted TGF-β-associated fibrosis, while antibiotic treatment attenuated these effects (Muraoka et al., 2023).

In contrast, Graciano-España et al. (2025) analyzed eutopic endometrium from 55 women with endometriosis and 37 controls and found no significant difference in *Fusobacterium* spp. abundance (P = .258) or *F. nucleatum* abundance (P = .738) (Graciano-España et al., 2025). Subgroup analyses by disease severity were also non-significant. Menstrual status was not uniform across groups; a higher proportion of endometriosis cases were menstruating at biopsy than controls, while participants were otherwise distributed across proliferative, ovulatory, secretory, and unknown phases. These findings do not replicate the strong tissue association reported by Muraoka et al. and underscore the current lack of reproducibility.

#### 3.3.2 Indirect non-tissue microbiome evidence

The two remaining studies provide indirect evidence because they evaluated microbiome compartments outside endometrial or endometriotic tissue. Hicks et al. (2025) studied 64 participants and found oral *Fusobacterium* enrichment among patients with moderate/severe endometriosis compared with minimal/mild disease, whereas vaginal and stool findings did not demonstrate an analogous *Fusobacterium* signal (Hicks et al., 2025). This anatomical specificity may indicate an association between oral microbial composition and disease severity, but it does not establish oral-to-reproductive-tract translocation or causality.

Jimenez et al. (2024) examined vaginal and rectal microbiomes in women with chronic pelvic pain with and without endometriosis and surgical controls. Among 73 participants in the final three-group comparison, *F. animalis*, historically classified as *F. nucleatum subsp. animalis*, was depleted in vaginal samples from the CPP-Endo group compared with CPP without endometriosis (LFC=-0.63) (Jimenez et al., 2024). No endometrial or endometriotic tissue was assessed. Thus, the Jimenez finding should not be described as *F. nucleatum*-specific tissue evidence.

Taken together, the direct tissue evidence is limited to two conflicting studies, while the other two reports provide indirect, anatomically distinct microbiome associations. No consistent pattern of *F. nucleatum* enrichment has been demonstrated across endometrial, oral, vaginal, rectal, and gastrointestinal compartments.

### 3.4 Risk of bias (quality) assessment

As shown in **Table 2**, all four human observational studies were judged to have an overall serious risk of bias, driven primarily by potential confounding. Adjustment or control for factors that could influence the microbiome, including antibiotic exposure, hormonal treatment, menstrual-cycle status, oral health, comorbid gynecologic conditions, and other clinical characteristics, was incomplete or inconsistent across studies. Participant selection, missing data, and selective reporting were generally considered at low risk. Classification of endometriosis status was predominantly at low risk because surgical and/or histopathological confirmation was reported. Muraoka et al. was rated Moderate in this domain because the study described surgical clinical cohorts but did not clearly report histopathological confirmation of endometriosis status for all participants contributing to the relevant human tissue analyses. Outcome measurement was rated Moderate across studies because of differences in microbial detection methods and potential contamination or taxonomic misclassification in low-biomass samples. Formal inter-rater agreement statistics were not calculated for the risk-of-bias judgments. The Serious overall ratings therefore reflect the most consequential domain-level concern - particularly confounding - and do not imply that all individual domains were equally problematic.

**Table 2:** Risk of bias assessment. Human observational studies (ROBINS-I adapted framework).

Study	Confounding	Selection	Classification	Missing	Outcome	Selective	Overall
Muraoka 2023 (Muraoka et al., 2023)	Serious	Low	Moderate	Low	Moderate	Low	Serious
Jimenez 2024 (Jimenez et al., 2024)	Serious	Low	Low	Low	Moderate	Low	Serious
Graciano-España 2025 (Graciano-España et al., 2025)	Serious	Low	Low	Low	Moderate	Low	Serious
Hicks 2025 (Hicks et al., 2025)	Serious	Low	Low	Low	Moderate	Low	Serious

Classification, classification of endometriosis status; Missing, missing data; Outcome, measurement of Fusoba*cterium*. Risk levels: Low, Moderate, Serious.

For the animal component of Muraoka et al. (2023), the SYRCLE assessment (**Table 3**) identified substantial uncertainty across most risk-of-bias domains. Sequence generation, baseline comparability, allocation concealment, random housing, performance blinding, random outcome assessment, and outcome-assessor blinding were rated as unclear because the relevant procedures were insufficiently reported. In contrast, incomplete outcome data and selective reporting were considered at low risk. The predominance of unclear ratings does not necessarily indicate that these methodological safeguards were absent, but rather that the available reporting was insufficient to determine whether they were adequately implemented. Consequently, the experimental findings provide important mechanistic evidence but should be interpreted with caution because of uncertainty regarding the internal validity of the animal experiments.

**Table 3:** Risk of bias assessment. Animal study (SYRCLE).

Study	Sequence	Base	Allocation	House	Blind1	Random	Blind2	Incomplete	Selective
Muraoka 2023 (Muraoka et al., 2023)	Unclear	Unclear	Unclear	Unclear	Unclear	Unclear	Unclear	Low	Low

Sequence, sequence generation; Base, baseline characteristics; Allocation, allocation concealment; House, random housing; Blind1, performance blinding; Random, random outcome assessment; Blind2, blinding of outcome assessors; Incomplete, incomplete outcome data; Selective, selectivereporting.

### 3.5 Certainty of evidence

The GRADE assessment, shown in **Table 4**, rated the certainty of evidence for all human observational outcomes as very low. For tissue-based *F. nucleatum* evidence, certainty was downgraded because of serious risk of bias, serious inconsistency between a strong positive tissue signal in the Muraoka subset and a null case-control result in Graciano-España, and serious imprecision. Non-tissue evidence was further downgraded for indirectness because oral, vaginal, rectal, and stool signals do not establish endometrial or lesion colonization.

**Table 4:** GRADE assessment of certainty of evidence.

Outcome	Studies	N	RoB	Inconsistency	Indirectness	Imprecision	Certainty
*F. nucleatum* detection/abundance in endometrial or endometriotic tissue	2	176	Serious	Serious	Not serious	Serious	Very Low
Non-tissue *Fusobacterium* abundance/enrichment in endometriosis	2	137	Serious	Serious	Serious	Serious	Very Low
Lesion promotion (mouse model)	1	N/A	Unclear	N/A	Serious	Not serious	Very Low
TGF-β pathway activation	1	N/A	Unclear	N/A	Serious	Not serious	Very Low

N, outcome-specific total participants in human studies; RoB, risk of bias.

### 3.6 Publication bias

Publication bias was not assessed because of the limited number of studies included in the review. With so few studies, conventional methods such as funnel plot inspection and statistical tests for funnel plot asymmetry would be unreliable and have insufficient power to meaningfully detect publication bias.

## 4 Discussion

### 4.1 Summary of evidence

This systematic review identified a small and heterogeneous body of evidence examining *Fusobacterium* in relation to endometriosis. The most direct human evidence comes from two tissue-based studies with conflicting results. Muraoka et al. (2023) reported frequent *Fusobacterium* detection in a defined subset of endometrial and endometriotic tissues from Japanese patients, whereas Graciano-España et al. (2025) found no significant difference in *F. nucleatum* abundance between 55 cases and 37 controls. The remaining studies provide indirect evidence from non-tissue microbial communities: Hicks et al. (2025) reported oral *Fusobacterium* enrichment among patients with moderate/severe endometriosis, while Jimenez et al. (2024) reported depletion of *F. animalis* in vaginal samples from women with chronic pelvic pain and endometriosis compared with chronic pelvic pain alone. Together, these findings do not demonstrate a consistent endometriosis-associated *F. nucleatum* signature across anatomical sites.

The strongest mechanistic evidence derives from Muraoka et al., whose experimental work showed that *F. nucleatum* promoted endometriotic lesion growth and fibrosis through TGF-β-associated pathways, with attenuation following antibiotic treatment (Muraoka et al., 2023). However, these findings have not been independently replicated, and none of the available human studies established that *F. nucleatum* exposure preceded endometriosis development. Accordingly, the overall certainty of evidence remains very low, and the available data support biological plausibility rather than a causal relationship.

### 4.2 Mechanistic plausibility

Despite inconsistent human detection, several biological mechanisms could plausibly link *F. nucleatum* to endometriosis. The organism can promote inflammatory and immune-modulatory responses and has been extensively investigated in colorectal cancer, where it contributes to a microenvironment favorable to inflammation, immune dysregulation, and disease progression (Kostic et al., 2013; Rubinstein et al., 2013). Similar processes could theoretically amplify the chronic inflammatory environment characteristic of endometriosis (Ghilea Seleș et al., 2026). Evidence from other gynecologic conditions also demonstrates that *F. nucleatum* can be detected in the female reproductive tract (Zheng et al., 2025); for example, Hakimjavadi et al. reported greater vaginal abundance in women with high-grade endometrial cancer than in those with benign gynecologic disease (Hakimjavadi et al., 2022). These observations support biological plausibility but do not establish that the organism originates from the oral cavity or contributes directly to endometriosis.

The TGF-β pathway provides a more specific potential mechanistic link. TGF-β is an important mediator of fibrosis (Wynn and Ramalingam, 2012), a prominent feature of endometriotic lesions, and promotes fibroblast-to-myofibroblast differentiation and extracellular matrix deposition (Vissers et al., 2024). Muraoka et al. demonstrated that *F. nucleatum* induced TGF-β1 secretion from endometrial epithelial cells and promoted myofibroblast differentiation *in vitro (*Muraoka et al., 2023). In their mouse model, bacterial inoculation increased lesion weight, collagen deposition, α-smooth muscle actin expression, and TGF-β pathway activation, whereas antibiotic treatment attenuated lesion growth and fibrosis (Muraoka et al., 2023). These findings provide a plausible pathway through which *F. nucleatum* could influence lesion progression.

Nevertheless, substantial uncertainty remains. The proposed pathway derives largely from a single experimental study, and the experimental inoculation model may not reproduce natural exposure or dissemination in humans. Furthermore, inconsistent detection of *F. nucleatum* in human endometrial tissue raises questions about whether it represents a reproducible component of the disease microenvironment. Thus, the experimental evidence should be regarded as hypothesis-generating until independently replicated and supported by longitudinal human studies. The proposed *Fusobacterium*–host–fibrosis axis is summarized in **Figure 2**.

**Figure 2:**
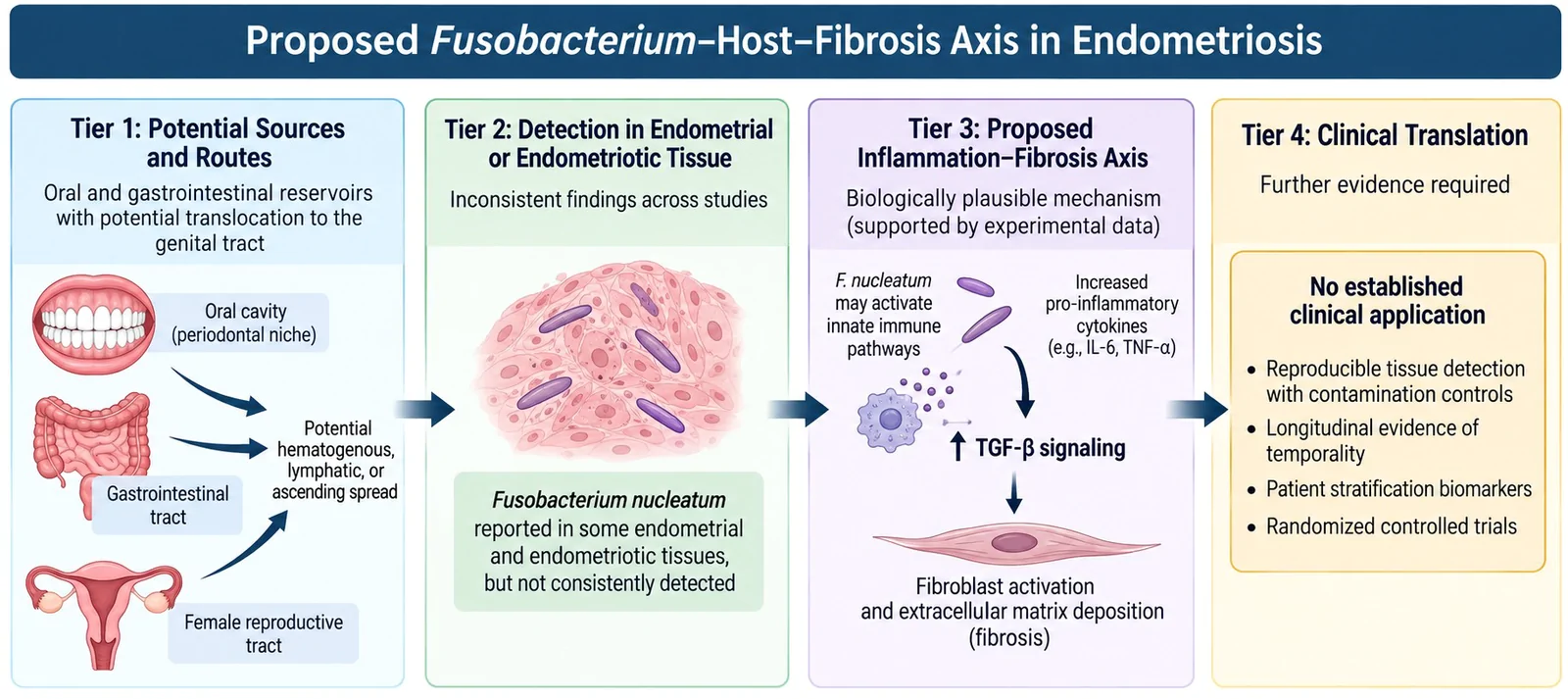
Proposed Fusobacterium-host-fibrosis axis in endometriosis. Conceptual framework illustrating the potential relationship between Fusobacterium nucleatum and endometriosis. Oral and gastrointestinal reservoirs represent possible sources of bacterial dissemination to the female reproductive tract through hematogenous, lymphatic, or ascending routes. Although *F. nucleatum* has been detected in some endometrial and endometriotic tissues, findings remain inconsistent across studies. Experimental evidence suggests that *F. nucleatum* may activate inflammatory pathways and increase pro-inflammatory cytokines and TGF-β signaling, promoting fibroblast activation, extracellular matrix deposition, and fibrosis. These mechanisms remain hypothesis-generating, and clinical translation will require reproducible tissue detection with appropriate contamination controls, evidence of temporality, patient-stratification biomarkers, and randomized controlled trials. Abbreviations: IL-6, interleukin-6; TGF-β, transforming growth factor beta; TNF-α, tumor necrosis factor alpha.

### 4.3 Methodological heterogeneity and inconsistent findings

Methodological heterogeneity is likely an important contributor to the divergent findings. The two tissue-based studies differed in population characteristics, disease phenotype, sampling procedures, menstrual status, DNA extraction and detection approaches, and molecular targets. Muraoka et al. enrolled 79 women with endometriosis and 76 controls in Japan; the principal FISH comparison included 42 cases and 42 controls and provided spatial localization of *Fusobacterium* signals in tissue. Graciano-España et al. analyzed 92 eutopic endometrial samples (55 cases and 37 controls) using genus- and species-specific qPCR and found no significant case-control difference. Their cohort was not restricted to the proliferative phase: participants were distributed across menstrual, proliferative, ovulatory, secretory, and unknown phases, with a significantly greater proportion of endometriosis cases menstruating at the time of biopsy. These differences may contribute to heterogeneity but do not by themselves explain the discordant findings.

Assay characteristics are also important. FISH provides spatial localization but taxonomic specificity depends on probe design and hybridization conditions. qPCR provides sensitive targeted detection but depends on primer specificity, extraction efficiency, and target selection. In contrast, 16S rRNA sequencing provides broader community profiling but may have limited species-level resolution depending on the sequenced region and analytical pipeline (Valido et al., 2025). Differences between targeted detection, presence/absence measurements, and relative-abundance approaches further limit direct comparison.

Anatomical sampling varied considerably. Muraoka et al. (2023) examined eutopic endometrium and endometriotic lesions, whereas Graciano-España et al. (2025) analyzed eutopic endometrium only. Hicks et al. (2025) examined oral, vaginal, and stool samples, and Jimenez et al. (2024) assessed vaginal and rectal microbiomes. Evidence at one site cannot be assumed to reflect bacterial presence within endometriotic lesions. This distinction is particularly important for the proposed oral microbe-host axis: oral enrichment provides evidence of association with the oral microbiome, but without paired strain-level analysis it cannot establish oral-to-reproductive-tract transmission (Li et al., 2026).

Contamination is an additional concern, particularly for low-biomass endometrial specimens. Reagent, environmental, and procedural contamination can generate microbial signals that may be difficult to distinguish from genuine tissue-associated bacteria. Inconsistent reporting of negative and procedural controls therefore reduces confidence in some findings. Geographic and population differences may also contribute through variation in microbiome composition, diet, oral health, medication exposure, age, disease stage, and other environmental or clinical factors (Ma et al., 2022; Govender and Ghai, 2025).

### 4.4 Clinical implications and antimicrobial stewardship

The current evidence does not support antibiotic treatment of endometriosis based on presumed *F. nucleatum* involvement. Although antibiotic treatment reduced lesion burden and fibrosis in the experimental model reported by Muraoka et al. (2023), these findings remain preclinical and have not been validated in randomized human trials. Moreover, inconsistent tissue detection suggests that even if a bacterial contribution exists, it may apply only to a subset of individuals.

Evidence supporting antibiotic treatment of chronic endometritis should not be extrapolated directly to endometriosis. Chronic endometritis is a distinct inflammatory condition for which antimicrobial treatment has been investigated in relation to reproductive outcomes (Kitaya et al., 2017). In contrast, a bacterial etiology for endometriosis has not been established. Unnecessary antimicrobial exposure may promote resistance and disrupt commensal microbial communities (Francino, 2015; Blaser, 2016). Therefore, antibiotics targeting *F. nucleatum* should not be recommended for endometriosis outside appropriately designed clinical trials.

### 4.5 Limitations

This review has several limitations. Most importantly, the evidence base was small, preventing quantitative meta-analysis and limiting confidence in the overall conclusions. The included studies had important risks of bias, particularly from confounding, and used markedly different biological compartments and microbial detection approaches. Outcome-specific denominators also differed from overall study enrollment in some reports, particularly Muraoka et al., requiring careful separation of study-level sample size from analysis-specific sample size. Methodological heterogeneity in study design, detection techniques, anatomical sampling, menstrual-cycle status, and contamination safeguards further limited direct comparison.

The review was restricted to published, peer-reviewed English-language studies and may therefore have missed relevant unpublished or non-English evidence. Although the original search included the genus-level term *Fusobacterium, F. animalis* was not listed as a separate free-text term, which could reduce sensitivity for records indexed only under the newer species name. In addition, the human risk-of-bias assessment used a prespecified adapted ROBINS-I framework rather than ROBINS-E. Because the current ROBINS-E tool is specifically designed for non-randomized follow-up studies estimating exposure effects, it was not substituted *post hoc* across this heterogeneous set of observational designs; nevertheless, future longitudinal etiologic reviews should preferentially consider ROBINS-E. A formal inter-rater agreement statistic was not calculated for risk-of-bias judgments. Finally, the principal mechanistic findings have not been independently replicated, and the geographic diversity of the small number of studies limits generalizability.

### 4.6 Future research directions

Future studies should prioritize standardized and contamination-aware approaches to determine whether *F. nucleatum* is reproducibly associated with endometriosis (Fierer et al., 2025). Sampling protocols should clearly define anatomical location, menstrual-cycle phase, collection procedures, recent antibiotic exposure, oral-health status, and appropriate negative and environmental controls. Combining targeted methods such as qPCR or FISH with sequencing-based approaches in the same specimens could help distinguish methodological discrepancies from genuine biological variation.

Testing the proposed oral microbe-host axis will require more than demonstrating *Fusobacterium* at separate anatomical sites. Prospective studies should collect paired oral samples - preferably saliva and subgingival plaque - together with eutopic endometrium and endometriotic lesions, and document periodontal status, oral hygiene, recent dental procedures, hormonal exposure, menstrual phase, and antibiotic exposure. Strain-level genomic approaches should then determine whether genetically related *F. nucleatum* strains occur in the oral cavity and reproductive tissues of the same individuals (Shimomura et al., 2023; Guan et al., 2025). Such studies would provide considerably stronger evidence for hematogenous or other routes of dissemination than genus- or species-level detection alone.

Longitudinal studies are also needed to establish temporality and determine whether *F. nucleatum* presence precedes lesion development, accompanies disease progression, or occurs secondary to the altered inflammatory environment of endometriosis. Independent replication of the proposed TGF-β-mediated mechanism in additional experimental models and human tissues is equally important. For future systematic reviews specifically evaluating longitudinal microbial exposure-effect relationships, ROBINS-E should be considered for risk-of-bias assessment (Higgins et al., 2024). Only if a reproducible bacterial association and clinically relevant mechanistic role are established should targeted antimicrobial interventions be evaluated in randomized controlled trials, with careful attention to patient selection, microbiome disruption, antimicrobial resistance, and long-term safety.

## 5 Conclusions

Current evidence suggests a biologically plausible but unconfirmed relationship between F. nucleatum and endometriosis. Four human studies encompassed 384 participants at the study level, although analysis-specific denominators varied. Crucially, direct tissue evidence comes from only two studies and is conflicting: one reported frequent *Fusobacterium* detection in endometrial/endometriotic tissue, whereas the other found no significant case-control difference in *F. nucleatum* abundance. The remaining studies provide indirect oral, vaginal, rectal, or stool microbiome evidence and cannot establish tissue colonization or translocation. All human studies were judged at serious overall risk of bias, and certainty of evidence was very low.

Experimental evidence suggests that *F. nucleatum* may promote lesion growth and fibrosis through TGF-β-associated pathways, but these findings derive primarily from a single translational study and have not been independently replicated. Thus, the proposed oral microbe-host axis remains hypothesis-generating rather than established. Future studies should use standardized contamination-controlled sampling, paired oral and reproductive-tract specimens, strain-level genomic analyses, and longitudinal designs to establish microbial origin, translocation, temporality, and causality. Until such evidence is available, *F. nucleatum* should not be considered an established etiologic or therapeutic target in endometriosis, and antimicrobial treatment for this purpose is not supported outside clinical trials.

## Data Availability

The original contributions presented in the study are included in the article/supplementary material. Further inquiries can be directed to the corresponding author.
